# Pathological findings of uterine tumors preoperatively diagnosed as red degeneration of leiomyoma by MRI

**DOI:** 10.1007/s00261-017-1126-3

**Published:** 2017-04-07

**Authors:** Go Nakai, Takashi Yamada, Takamitsu Hamada, Natsuko Atsukawa, Yoshikazu Tanaka, Kiyohito Yamamoto, Akira Higashiyama, Hiroshi Juri, Atsushi Nakamoto, Kazuhiro Yamamoto, Yoshinobu Hirose, Masahide Ohmichi, Yoshifumi Narumi

**Affiliations:** 1 Department of Radiology, Osaka Medical College, 2-7 Daigaku-machi, Takatsuki, Osaka 569-8686 Japan; 2 The Department of Pathology, Osaka Medical College, 2-7 Daigaku-machi, Takatsuki, Osaka 569-8686 Japan; 3 Department of Obstetrics and Gynecology, Osaka Medical College, 2-7 Daigaku-machi, Takatsuki, Osaka 569-8686 Japan

**Keywords:** Red degeneration, MRI, Pathological finding, Leiomyoma, Imaging

## Abstract

**Purpose:**

Venous infarction of a leiomyoma is known as red degeneration of leiomyoma (RDL) and can be a cause of acute abdomen. Although magnetic resonance imaging (MRI) is the only modality that can depict the inner condition of a leiomyoma, the typical MR findings of RDL are sometimes identified incidentally even in asymptomatic patients. The purpose of this study is to clarify common pathological findings of uterine tumors preoperatively diagnosed as RDL by MRI.

**Methods:**

We diagnosed 28 cases of RDL by MRI from March 2007 to April 2015. The ten lesions subjected to pathological analysis after resection were included in the study and reviewed by a gynecological pathologist. The average time from MRI to operation was 4.7 months.

**Results:**

The typical beefy-red color was not observed on the cut surface of the tumor except in one tumor resected during the acute phase. All lesions diagnosed as RDL by MRI had common pathological findings consistent with red degeneration of leiomyoma, including coagulative necrosis. Other common pathological features of RDL besides extensive coagulative necrosis appear to be a lack of inflammatory cell infiltrate or hemorrhage in the entire lesion.

**Conclusions:**

Although RDL is known to cause acute abdomen, its typical MR findings can be observed even in asymptomatic patients in a condition that manifests long after red degeneration. The characteristic pathological findings in both the acute phase and the chronic phase that we found in this study, along with radiology reports, will be helpful references for gynecologists and pathologists in suspecting a history of red degeneration and confirming the diagnosis.

Red degeneration of leiomyoma (RDL) is believed to be caused by sudden venous obstruction of the leiomyoma and is often associated with pregnancy or the use of contraceptive drugs [1]. The name “red degeneration” has been well known among radiologists since its appearance on magnetic resonance imaging (MRI) was first described in 1994 [2], but it is virtually unknown to gynecologists and pathologists. One reason is that gynecologists rarely perform MRI on patients with leiomyoma who complain of low abdominal pain localized to the lesion because it is a common complaint and most patients respond to supportive measures performed in accordance with a clinical diagnosis of leiomyoma degeneration [3]. Therefore, very few patients with RDL undergo surgery during the acute phase, which means that pathologists have fewer opportunities to observe its typical gross and microscopic appearance. A few pathology textbooks show gross features such as a bulging surface and homogeneous dark red appearance, but none show its detailed microscopic appearance, instead explaining it in words as “extensive coagulative necrosis” [1, 4]. It is also not currently classified as a leiomyoma variant in the WHO classification [5]. The aim of this study is to describe common pathological findings of uterine tumors preoperatively diagnosed as RDL by MRI.

## Materials and methods

In our hospital, 28 cases of RDL were diagnosed by MRI from March 2007 to April 2015. These cases were retrieved from the radiology report database using the keyword “red degeneration of leiomyoma.” Common MRI findings were as follows. All uterine lesions were well demarcated and had a hyperintense rim or were entirely hyperintense compared to uterine myometrium on T1-weighted imaging (WI). On T2WI, the lesions had a distinct hypointense rim or were entirely hypointense compared to uterine myometrium. In three cases (cases 1, 5, and 9) in which gadolinium-enhanced fat-suppressed T1WI was performed, the tumors did not show any contrast enhancement. These findings are known among radiologists as the MR appearance of red degeneration of uterine leiomyoma [2]. A radiologist (G.N.) with 12 years of experience in gynecological imaging confirmed that all lesions met the criteria. MR findings in each patient are summarized in Table 1.

**Table 1:** MR findings in each patient

Case	Age	T1WI	T2WI	DWI	Gadolinium-enhanced fat-suppressed T1WI
1	41	Hypointense with hyperintense rim	Heterogeneous hypointense with distinct hypointense rim	Isointense with thin, distinct hypointense peripheral rim	Complete absence of tumor contrast enhancement
2	44	Slightly hyperintense	Hyperintense	Hypointense	N/A
3	35	Inhomogeneous hyperintense	Heterogeneous hypointense with a distinct hypointense rim	Hyperintense	N/A
4	34	Hyperintense	Hyperintense with a thick hypointense rim	Heterogeneous hyperintense	N/A
5	45	Hypointense with hyperintense rim	Hypointense	Hypointense	Complete absence of tumor contrast enhancement
6	39	Hyperintense	Hyperintense with distinct hypointense rim	N/A	N/A
7	47	Mildly hyperintense	Heterogeneous hypointense with distinct hypointense rim	Heterogeneous hyperintense with distinct hypointense rim	N/A
8	31	Hypointensewith hyperintense rim	Hypointense	N/A	N/A
9	34	Mildly hyperintense	Hyperintense with distinct hypointense rim	Hyperintense with distinct hypointense rim	Complete absence of tumor contrast enhancement
10	33	Slightly hyperintense with distinct hyperintense rim	Hypointense with distinct hypointense rim	N/A	N/A

Signal intensity (SI) shown is SI relative to SI of uterine myometrium

*N/A* not available

MRI was performed with a 1.5-T superconducting magnet (Signa HDxt or Signa Excite; GE Healthcare, Milwaukee, WI) using a phased array coil. Axial and sagittal fast spin echo T2WI were acquired with a repetition time (TR) range/echo time (TE) range of 3000–5000/69–110 ms, a 5–6 mm slice thickness/1–3 mm interslice gap, and a 256 × 192–320 × 224 matrix. Axial T1WI were acquired with a spin echo TR range/TE range of 135–600/4.2–14 ms, a 5–6 mm slice thickness/1–3 mm interslice gap, and a 256 × 192–320 × 224 matrix. These sequences were obtained for all patients. Gadolinium-enhanced fat-suppressed T1WI and diffusion-weighted single-shot echo-planar images (DWIs) obtained with b values of 0 and 800 ms/mm^2^ were acquired in the axial plane in some patients.

Eleven patients underwent hysterectomy or myomectomy during the course of clinical follow-up. One patient was excluded because she underwent surgery for bilateral ovarian borderline tumors. Consequently, the ten patients without malignant diseases were included in this study. All histological slides were reviewed by a gynecological pathologist (T.Y.) with 30 years of experience to find common pathological findings of lesions diagnosed as RDL by radiologists. All lesions were embedded in paraffin and stained with hematoxylin and eosin. Clinical courses of patients as well as radiological and pathological characteristics of the lesions were investigated by reference to each clinical record and MR images.

There is no clearly established definition of the acute phase of RDL, but it is defined as within 1.5 months after onset in this study.

## Results

Clinical courses of patients are summarized in Table 2. The mean patient age was 38 years (range 31–47). The average time from MRI to operation was 4.7 months (range 1.2–16) and 3 patients (cases 2, 3 and 10) were treated with gonadotrophin-releasing hormone agonists before surgery. Past symptoms suggestive of red degeneration were mentioned in clinical records of 6 patients (cases 1, 3, 4, 8, 9 and 10), all of whom experienced low abdominal pain with or without fever and were treated with a pain reliever and antibiotics in accordance with a clinical diagnosis of endometritis or myometritis made without using MRI. The suspected risk factor for onset was postpartum status for two patients (cases 4 and 8), post-abortion status for one patient (case 3) and pregnancy for two patients (cases 9 and 10). One patient (case 1) did not have any risk factors. Symptoms suggestive of red degeneration were not found in past clinical records of any other patients. Four patients underwent hysterectomy, five patients underwent laparoscopic myomectomy using a power morcellator (cases 3, 4, 6, 8, and 10), and one patient underwent abdominal myomectomy. A power morcellator was used before the U.S. Food and Drug Administration recommended strict limits on use of the tool for fibroid surgery in 2014. Radiological and pathological characteristics of the lesions are summarized in Table 3. Four patients had multiple myomas, but only one myoma was diagnosed as red degeneration on MRI in each patient (cases 2, 4, 5, and 7). On MRI, all lesions were well demarcated and ranged in size from 4.3 to 10.8 cm in largest diameter with a mean of 7.6 cm. The location of the affected leiomyoma was intramural in eight patients and submucosal in two patients.

**Table 2:** Clinical courses of patients

Case	Age	Symptom suggestive of RL	Suspected risk factor for the onset	Treatment before surgery	The period^a^ between the onset and operation (M)	The period^a^ between the last MRI and surgery (M)	The reason for the operation	Surgery
1	41	LAP, fever	Unclear	6 month course of GnRHa	1.4	1.2	Intractable fever	ATH
2	44	Unclear	Unclear	3 month course of GnRHa	N/A	2	Abnormal genital bleeding	TLH
3	35	LAP, fever	Post-abortion	None	6	4.8	Abortion coused by leiomyoma supposedly	LM using a PM
4	34	LAP	Postpartum	None	4.2	2.1	Dysuria, LAP and an ovarian tumor	LM using a PM
5	45	Unclear	Unclear	None	N/A	15.8	Pollakiuria, low abdominal protuberance	ATH
6	39	Unclear	Unclear	None	>36	2	Dysmenorrhea	LM using a PM
7	47	Unclear	Unclear	None	N/A	3.8	Hypermenorrhea, abnormal genital bleeding	ATH
8	31	LAP	Postpartum	None	2.3	1.9	Abnormal genital bleeding	LM using a PM
9	34	LAP	During pregnancy	None	19.5	5.7	Large uterin tumor and an ovarian tumor	Abdominal myomectomy
10	33	LAP	During pregnancy	5 month course of GnRHa	>34	8	Large uterin tumor	LM using a PM

^a^Period is in months (M)

*LAP* low abdominal pain, *RDL* red degeneration of leiomyoma, *ATH* abdominal total hysterectomy, *TLH* total laparoscopic hysterectomy, *LM* laparoscopic myomectomy, *PM* power morcellator

**Table 3:** Radiological and pathological characteristics of lesions

Case	The location of the affected myoma	The number of the affected myoma	The total number of myoma	The maximum diameters of the affected myoma	Postoperative pathological report	Gross appearance	Coexisting disease
1	Intramural	1	1	108	Degenerative leiomyoma	Beefy-red appearance	None
2	Intramural	1	5	43	Leiomyoma	Homogeneous pink-tan appearance	None
3	Intramural	1	1	70	Hyaline degeneration of leiomyoma	Homogeneous pink-tan appearance	None
4	Intramural	1	2	76	Leiomyoma	Homogeneous pink-tan appearance	Mature cystic teratoma of the right ovary
5	Intramural	1	More than 20	71	Leiomyoma	N/A	None
6	Intramural	1	1	54	Hyaline degeneration of leiomyoma or coaculative necrotic tissue	Homogeneous pink-tan appearance	None
7	Intramural	1	2	80	Hyaline degeneration of leiomyoma	Homogeneous pink-tan appearance	None
8	Submucosal	1	1	62	Necrotic tissue	Homogeneous pink-tan appearance	None
9	Submucosal	1	1	101	Hyaline degeneration of leiomyoma	Homogeneous yellow-tan appearance	Endometriotic cyst of the left ovary
10	Intramural	1	1	97	Degenerative leiomyoma	Homogeneous pink-tan appearance	None

The location, number, and maximum diameter of lesions was assessed by MRI

Postoperative pathological diagnoses at that point were hyaline degeneration, coagulative necrosis, or degenerative leiomyoma. There was no definite pathological diagnosis of RDL. Two cases were also associated with benign ovarian tumors (cases 4 and 9).

**Fig. 1:**
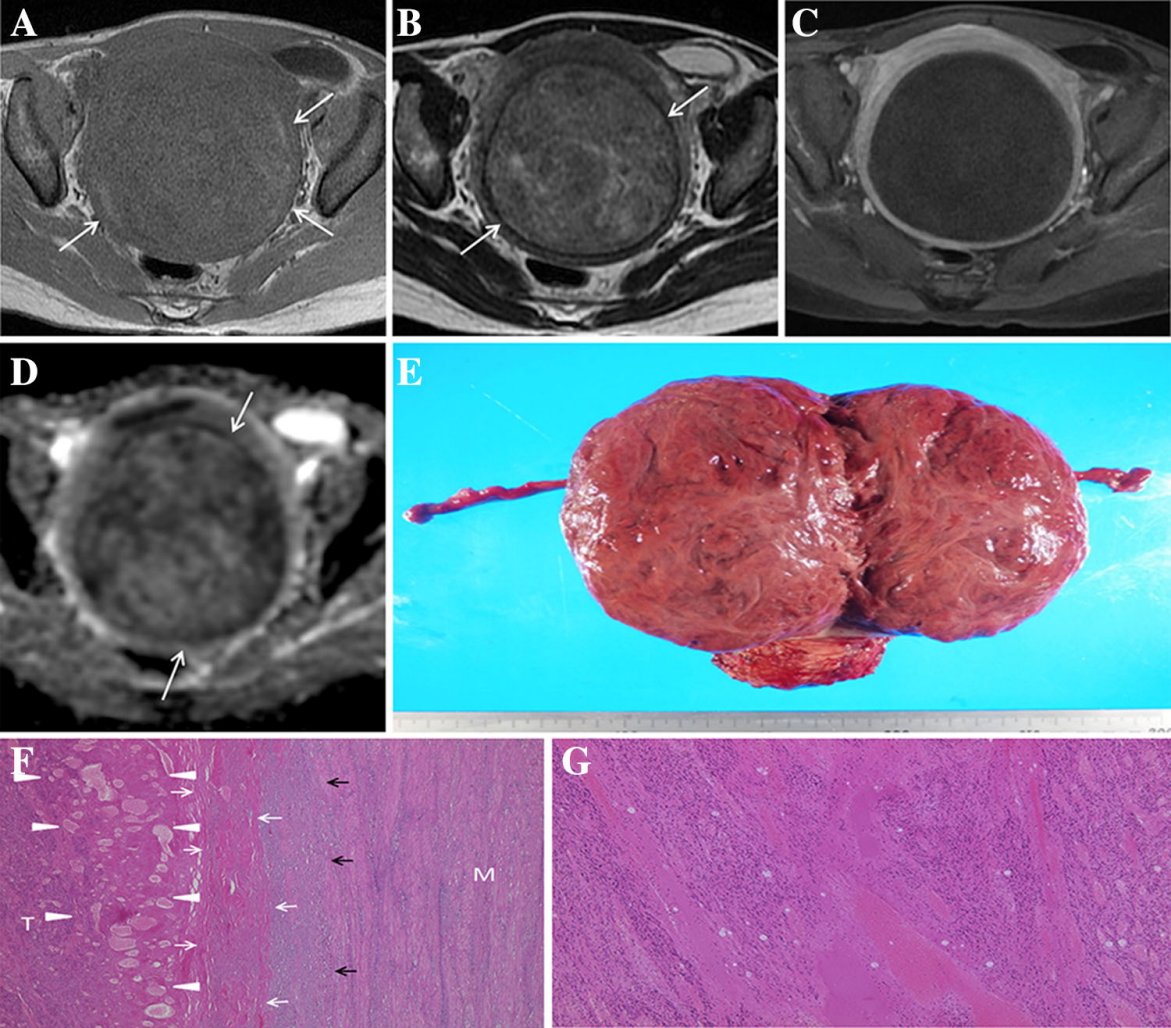
Case 1. 41-year-old woman who underwent surgery 1.4 months after onset. **A** Axial T1-weighted image shows a thin peripheral hyperintense rim surrounding the lesion’s central area of lower signal intensity. However, the rim is not concentric (*arrows*). **B** Axial T2-weighted image shows the lesion, accompanied by a distinct hypointense rim (*arrows*), exhibiting inhomogeneous low signal intensity. **C** Contrast-enhanced T1-weighted image shows complete absence of tumor contrast enhancement. **D** ADC map shows inhomogeneous restricted diffusion within the tumor accompanied by a thin, distinct hypointense peripheral rim (*arrows*). **E** Gross-cut surface of the hysterectomy specimen shows the typical *beefy-red color*. **F** Photomicrograph of the peripheral portion of the tumor shows a continuous band of tissue, representing the prompt appearance of complete coagulative necrosis (*white arrows*). A band of inflammatory cell infiltrate is observed around the tumor (*black arrows*), but inflammatory cells were not detected inside the tumor. Many dilated vessels are visible at the periphery of the tumor (*arrowheads*), indicative of its venous infarction. *T* tumor, *M* uterine myometrium. **G** Photomicrograph of the central portion of the tumor shows hypereosinophilia and various degrees of nuclear pyknosis without hemorrhage

**Fig. 2:**
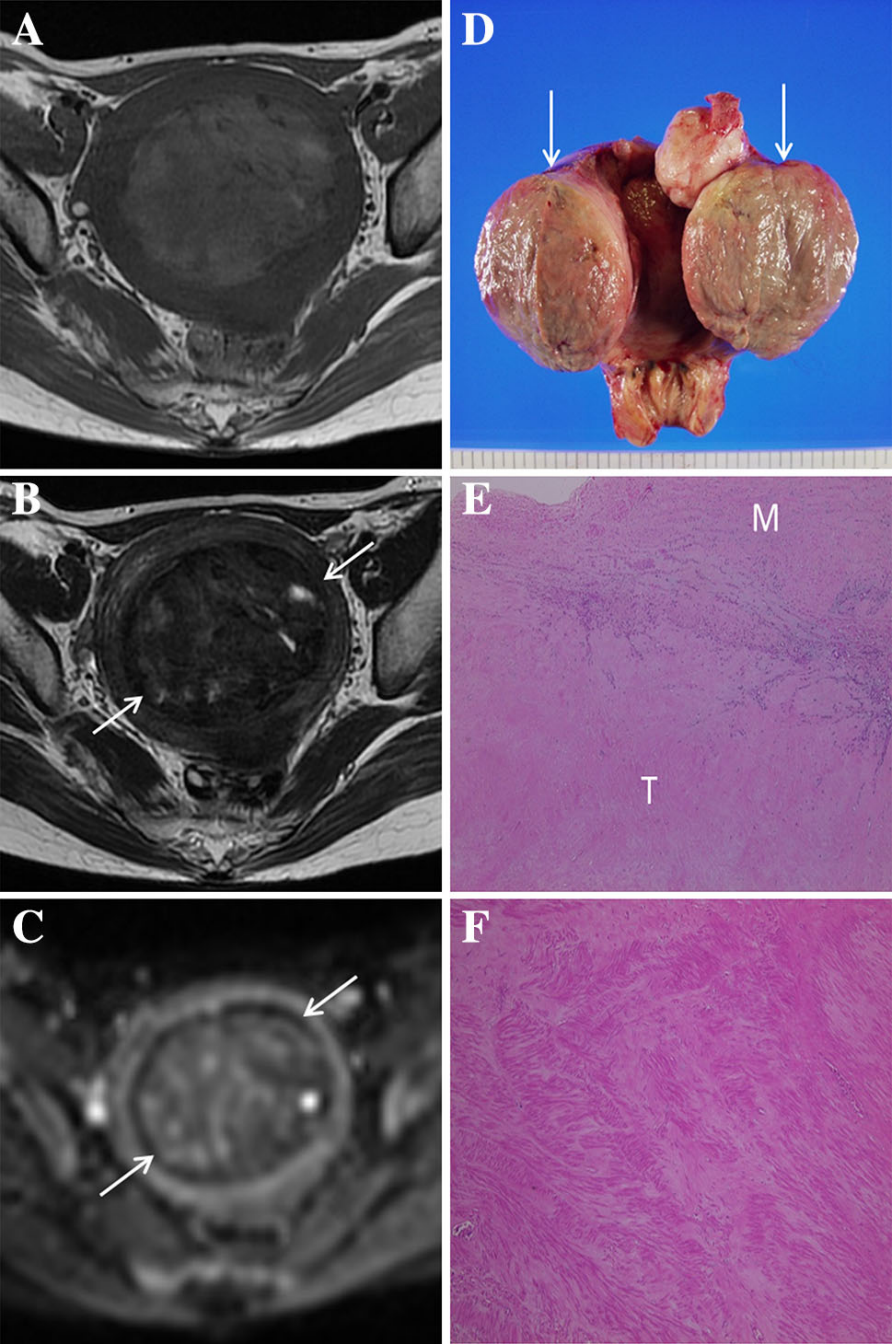
Case 7. 47-year-old woman. Symptoms suggestive of red degeneration were not mentioned in the clinical report. Underwent surgery 3.8 months after last MRI. **A** Axial T1-weighted MR image shows an entirely hyperintense lesion compared to myometrium. **B** Axial T2-weighted image shows the lesion, accompanied by a distinct hypointense rim (*arrows*), exhibiting inhomogeneous low signal intensity. **C** ADC map shows inhomogeneous restricted diffusion within the tumor accompanied by a thin, distinct hypointense peripheral rim (*arrows*). **D** Gross-cut surface of the hysterectomy specimen shows homogeneous pink-tan appearance throughout the lesion (*arrows*). **E** Dilated vessels are not identified at the periphery of the tumor. *T* tumor, *M* uterine myometrium. **F** Photomicrograph of the central portion of the tumor shows loss of myocyte nuclei but preserved myocyte striations, indicating coagulative necrosis. Inflammatory cell infiltrate is not identified, and thus the tumor lacks hemosiderin, granulation and fibrosis

**Fig. 3:**
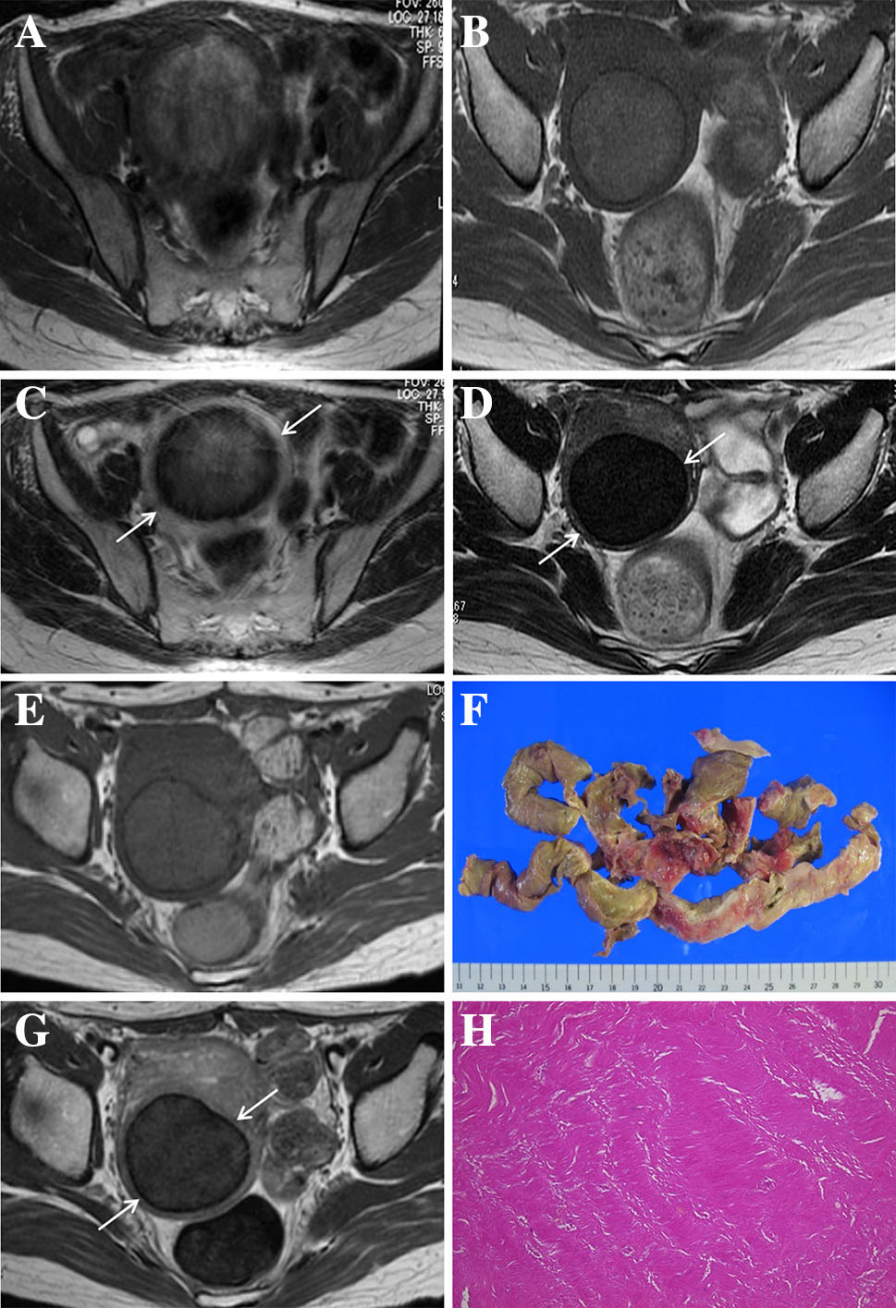
Case 6. 39-year-old woman. Symptoms suggestive of red degeneration were not mentioned in the clinical report. Although the time of symptom onset was not estimated precisely, she underwent MRI three times from 2008 through 2011 and typical radiological findings of red degeneration on MRI were observed as early as 2008 (**A** T1-weighted image, **B** T2-weighted image). **A** Axial T1-weighted image shows an entirely hyperintense lesion compared to myometrium. **B** Axial T2-weighted image shows the lesion, accompanied by a distinct hypointense rim (*arrows*), exhibiting slightly high signal intensity. The lesion is entirely hyperintense compared to myometrium on T1-weighted images, even in 2010 and 2011 (**C** and **E**, respectively), although the signal intensity in those years is less than that in 2008. A thin, distinct hypointense peripheral rim (*arrows*) is also identified on T2-weighted images in 2010 and 2011 (**D** and **F,** respectively). The signal intensity of the lesion on T2-weighted images in 2011 (**F**) is lower than that in 2010 (**D**). **G** Gross examination of the lesion resected by laparoscopic myomectomy using a power morcellator shows a homogeneous pink-tan appearance. **H** Photomicrograph of the lesion shows coagulative necrosis similar to that shown in Fig. 2F

Common pathological findings were (1) coagulative necrosis, in which the architecture of ghost cells is preserved, (2) lack of inflammatory cell infiltrate, (3) lack of hemosiderin in the tumor, (4) lack of hemorrhagic necrosis, and (5) that these pathological changes were seen extensively across the entire lesion. These findings were observed regardless of the time between MRI and surgery. The typical beefy-red color in gross appearance was observed as a macroscopic finding in only one patient, who underwent surgery just 1.4 months after onset (case 1) (Fig. 1). The typical appearance was not observed in other patients who underwent surgery more than 2.3 months after onset. Most lesions whose gross-cut surface was observed after resection demonstrated a homogeneous pink-tan appearance throughout (Table 3, Figs. 2, 3). Photographs of the hysterectomy specimen were available in case 5, but were not used to identify the cut surface of the tumor.

## Discussion

The MR appearance of acute-phase red degeneration of uterine leiomyoma was described in a study in which all five cases were associated with abortion or pregnancy [2]. In this study, five of six patients who had symptoms suggestive of red degeneration had those risk factors, but the remaining patient did not have any risk factors (case 1).

In the previous study, a hyperintense rim on T1WI and hypointense rim on T2WI were found to be the common MR appearance of RDL [2, 6]. However, RDL sometimes appears incidentally in asymptomatic patients as a well demarcated tumor that is entirely hyperintense compared to the myometrium on T1WI and shows heterogeneous, predominant hypointensity compared to the uterine myometrium on T2WI. Those tumors have generally also been diagnosed as RDL by radiologists, but their gross and microscopic appearances have never been investigated before.

Interestingly, all lesions in this study demonstrated common pathological features, mainly showing extensive coagulative necrosis without inflammatory cell infiltrate or hemorrhage throughout the entire lesion. A few studies explain that the microscopic appearance of red degeneration shows the ghosts of the muscle cells and their nuclei, or extensive coagulative necrosis [6, 7]. Although red degeneration appears similar to myocardial infarction in that coagulative necrosis of muscle cells manifests as deeply eosinophilic cytoplasm and loss of nuclei [8], the difference between them is that red degeneration lacks inflammatory cell infiltration, and thus also lacks hemosiderin, granulation and fibrosis.

The high signal intensity on T1WI is likely secondary to the proteinaceous content of the blood or the T1-shortening effects of methemoglobin [9, 10]. One patient did not undergo surgery for more than 3 years after onset (case 6) (Fig. 3). Although symptoms suggestive of red degeneration were not mentioned in her medical records, she underwent MRI three times from 2008 through 2011. Typical radiological findings of red degeneration on MRI were already observed in 2008, and the lesion was entirely hyperintense compared to the myometrium on T1WI even in 2010 and 2011 although the signal intensity in those years was less than that in 2008. This change in signal intensity on T1WI implies that cytoplasm of smooth muscle cells with coagulative necrosis itself also shows hyperintensity on T1WI because the numerous dilated vessels filled with red blood cells that were considered the cause of hyperintensity on T1WI in acute-phase patients [2] were not found microscopically in chronic-phase patients. These MR and microscopic findings observed in the chronic phase seem to be very similar to a case introduced as a leiomyoma with coagulative necrosis in previous article [10]. This type of leiomyoma is assumed to be a condition that manifests long after red degeneration. Peripheral dilated vessels were found in only one patient who underwent surgery during the acute phase, and the necrosis developed from the outside to the inside of the tumor (Fig. 1F). Therefore, dilated vessels within the lesion at the periphery might be a temporal change that can be observed only during the acute phase. More importantly, the lack of hemorrhagic necrosis indicates that its hyperintensity on T1WI is not caused by hemorrhage.

Only one patient underwent surgery during the acute phase, and the reason was that intractable fever had not resolved after more than 1 month of pharmacotherapy. Red degeneration can be associated with systemic symptoms such as low abdominal pain, pyrexia and leukocytosis [11]. In this study, a band of inflammatory cell infiltrate observed around a leiomyoma with red degeneration during the acute phase might be a cause of pyrexia and leukocytosis, although no inflammatory cells were detected inside the tumor (Fig. 1F). The other patients underwent surgery due to intolerable symptoms of uterine myoma such as dysuria, pollakiuria, dysmenorrhea or hypermenorrhea, or due to coexisting ovarian masses. In these patients who underwent surgery during the chronic phase, it was not easy for pathologists to recognize the history of red degeneration by its gross appearance because the typical dark red color was not observed on the cut surface of the tumor and most of them showed an entirely homogeneous pink-tan cut surface that could be found in necrosis (Figs. 2D, 3G). Such a gross appearance might make pathologists and gynecologists suspect that the mass might not be benign. Therefore, they should be aware of the preoperative diagnosis by MRI, as MRI is the only imaging modality that can detect RDL.

Other post-operative pathology findings besides coagulative necrosis that were described in reports were hyaline degeneration and degenerative leiomyoma. However, these were revealed to be an imprecise description after all of the pathological specimens were reviewed by a gynecological pathologist because coagulative necrosis was commonly observed in all lesions. Some explanations for the discrepancy between the original pathology reports and the histology after review by a gynecological pathologist in this study could be that it is not easy for pathologists to correctly diagnose RDL because very few patients with RDL undergo surgery during the acute phase, and thus pathologists have fewer opportunities to observe its typical gross and microscopic appearance and that pathologists do not know to infer a history of red degeneration when they encounter a uterine smooth muscle tumor with extensive coagulative necrosis.

The present study has several limitations. The first is that there was a long interval between MR examinations and surgery. However, MRI was used to identify patients with suspected RDL or a history of RDL. Acute-phase RDL was defined as within 1.5 months after onset for pathological analysis in this study. Hence, lesions in all patients other than patient 1 were determined to be chronic-phase RDL pathologically. Another limitation is the small size of the study population.

In conclusion, all lesions diagnosed as RDL by MRI had common pathological findings consistent with red degeneration of leiomyoma, including coagulative necrosis. Other common pathological features of RDL besides extensive coagulative necrosis appear to be a lack of inflammatory cell infiltrate or hemorrhage in the entire lesion. Although RDL is known to cause acute abdomen, its typical MR findings can be observed even in asymptomatic patients in a condition that manifests long after red degeneration. The characteristic pathological findings in both the acute phase and the chronic phase that we found in this study, along with radiology reports, will be helpful references for gynecologists and pathologists in suspecting a history of red degeneration and confirming the diagnosis.
